# Toward Value-Based Healthcare through Interactive Process Mining in Emergency Rooms: The Stroke Case

**DOI:** 10.3390/ijerph16101783

**Published:** 2019-05-20

**Authors:** Gema Ibanez-Sanchez, Carlos Fernandez-Llatas, Antonio Martinez-Millana, Angeles Celda, Jesus Mandingorra, Lucia Aparici-Tortajada, Zoe Valero-Ramon, Jorge Munoz-Gama, Marcos Sepúlveda, Eric Rojas, Víctor Gálvez, Daniel Capurro, Vicente Traver

**Affiliations:** 1 SABIEN-ITACA, Universitat Politècnica de València, 46022 València, Spain; geibsan@itaca.upv.es (G.I.-S.); anmarmil@itaca.upv.es (A.M.-M.); luaptor@itaca.upv.es (L.A.-T.); zoevara@itaca.upv.es (Z.V.-R.); vtraver@itaca.upv.es (V.T.); 2 Unidad Mixta de Reingeniería de Procesos Sociosanitarios (eRPSS), Instituto de Investigaciń Sanitaria del Hospital Universitario y Politecnico La Fe, Bulevar Sur S/N, 46026 València, Spain; 3 Hospital General de Valencia, Av. de les Tres Creus, 2, 46014 València, Spain; angelescelda@gmail.com (A.C.); mandingorra_jes@gva.es (J.M.); 4 School of Nursing, Universidad Católica de Valencia, 46022 València, Spain; 5 School of Engineering, Pontificia Universidad Católica de Chile, Santiago 8320000, Chile; jmun@ing.puc.cl (J.M.-G.); marcos@ing.puc.cl (M.S.); vagalvez@uc.cl (V.G.); 6 School of Medicine, Pontificia Universidad Católica de Chile, Santiago 8320000, Chile; eric.rojas@uc.cl (E.R.); dcapurro@med.puc.cl (D.C.)

**Keywords:** process mining, stroke, emergency, value-based healthcare, interactive

## Abstract

The application of Value-based Healthcare requires not only the identification of key processes in the clinical domain but also an adequate analysis of the value chain delivered to the patient. Data Science and Big Data approaches are technologies that enable the creation of accurate systems that model reality. However, classical Data Mining techniques are presented by professionals as black boxes. This evokes a lack of trust in those techniques in the medical domain. Process Mining technologies are human-understandable Data Science tools that can fill this gap to support the application of Value-Based Healthcare in real domains. The aim of this paper is to perform an analysis of the ways in which Process Mining techniques can support health professionals in the application of Value-Based Technologies. For this purpose, we explored these techniques by analyzing emergency processes and applying the critical timing of Stroke treatment and a Question-Driven methodology. To demonstrate the possibilities of Process Mining in the characterization of the emergency process, we used a real log with 9046 emergency episodes from 2145 stroke patients that occurred from January 2010 to June 2017. Our results demonstrate how Process Mining technology can highlight the differences between the flow of stroke patients compared with that of other patients in an emergency. Further, we show that support for health professionals can be provided by improving their understanding of these techniques and enhancing the quality of care.

## 1. Introduction

There is increasing interest in the optimization of healthcare in order to obtain a sustainable delivery of care [1,2]. In the healthcare domain, there is a growing emphasis on the importance of novel paradigms, such as Value-Based healthcare [2], which is based on maximizing the value delivered to the patient and Triple Aim [1], which defines value not only from a cost-effectiveness point of view but also from a quality of care perspective by taking into account the experience of the patient and the Quality of health. However, the application of these standards is not an easy task. To successfully apply these paradigms, it is necessary to provide technical solutions that enable professionals to improve their understanding of the value chain in health processes in order to support them in the application of adequate health policies.

Big Data and Data Science technologies are regarded as having a key role in the extraction of information from existing databases, personal devices and other information sources to support health professionals in their daily decisions [3]. The analysis of the large amount of data available in hospitals that can be used to support the optimization of clinical processes is an ongoing challenge [4]. These technologies enable health professionals in the creation of better care processes for the improvement of the Quality of Care provided to patients, the effectiveness of the treatments and their cost-effectiveness to patients and such improvements will enhance the sustainability of the health system [5]. Improvements in clinical process management not only save lives but also support the provision of better and personalized care to more patients.

However, typical Data Science solutions are shown as black boxes by professionals. This affects the confidence of professionals who are suspicious of the results offered by these technologies. In addition, these inductive methods are based on statistical frameworks that produce accurate results only when the number of cases is adequate. However, clinicians are unlikely to need more support in the standard case because is usually covered by standard treatment. Clinicians require help with treating rare cases and classic Data Science technologies have poorer accuracy in these contexts. An interactive methodology [6] is intended to provide a solution to this problem. This methodology involves experts who are in the middle of the learning process and, therefore, becoming aware of the real process. This increases the confidence of the user and speeds up the learning process by incorporating the knowledge being acquired by the expert in the inferred model [6]. Nevertheless, interactive models require Human-understandable Data Science models to support professionals during each learning iteration. Process Mining [7] solutions can facilitate health professionals’ understanding of the real clinical process of emergencies. Process Mining uses machine learning technologies to infer and analyze flows in a human-understandable manner [8].

Interactive Models have been applied to Process Mining in previous works using a question-driven approach [9]. The aim of this paper is to evaluate the ability of Process Mining to analyze the flow of emergencies in a hospital. For this purpose, clinical processes for stroke patients are analyzed using a Question-Driven methodology. Using this idea, we presented our preliminary work in Reference [10], in which we presented the following questions:

- Q1: Can Process Mining detect and measure the special characteristics of stroke emergency processes?
- Q2: Is Process Mining able to measure organizational changes that affect the emergency process?

In this paper, we extend our previous work by adding three more questions:

- Q3: Can Process Mining reveal differences in Emergency process protocols depending on Patients’ personal characteristics?
- Q4: Can Process Mining evaluate the status of the Emergency protocol according to existing Gold Standards?
- Q5: Can Process Mining provide a Healthcare Value-Based view of the effects of the care provided?

These questions were formed on the basis of the main concepts of the Value-Based Healthcare and Triple Aim paradigms in order to show the potential of Process Mining in this field. Specifically, this work is intended to study stroke cases and their characteristics. To this end, we analyzed a real log of 9046 Emergency episodes of 2145 patients who suffered at least one stroke event between January 2010 and June 2017. This log was used to answer the posed questions using process mining technologies and measure the statistical significance of the results.

The remainder of the paper is as follows. First, the related work section presents more details of the Value-Based Healthcare paradigm and Process Mining technologies are described in more detail. Then, the PMApp Tool and Statistical significance maps for supporting the confidence of professionals are described. After that, the results of the proposed methodology and selected experiments that address the research questions are explained. Finally, a discussion of the results concludes the paper.

## 2. Related Work

Since the inception of the Evidence-Based Medicine (EBM) Paradigm [11], there have been continuous attempts to gather evidence that supports Clinical professionals in their daily practice. As a result of this paradigm, important advances in healthcare and medicine have been achieved through the improvement of research methodologies. However, some authors have asserted their opposition to the excessive methodological focus of EBM [12]. Proponents of recent paradigms argue for new personalized ways to deliver care instead of the constant use of standardized protocols [13]. Other authors have claimed that taking into account the Values that affect evidence is more important than focusing on methods [14]. The Value-Based Healthcare (VBHC) paradigm has arisen from these criticisms of EBM [2].

### 2.1. Toward Value-Based Healthcare delivery in the Emergency Process

**Figure 1:**
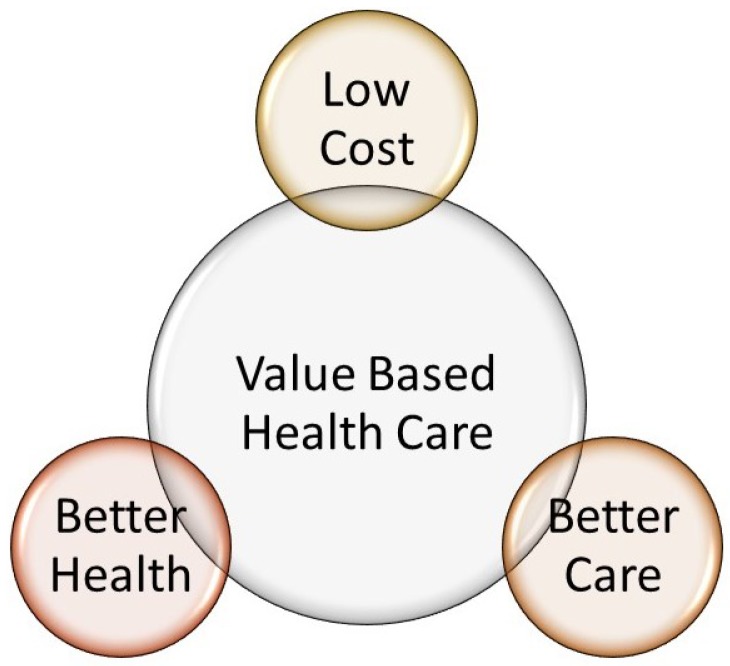
Value-based Healthcare and Triple Aim paradigms.

The Value-Based Healthcare paradigm aims to deliver healthcare with maximized benefits that are transferred to the patient by selecting the best options according to the evidence while considering the value chain. In other words, the objective of VBHC is to get the best outcome for the patient using as few resources as possible [15]. Beyond this idea, the Triple Aim paradigm [1] goes a step further by proposing three main concepts called *aims* to define the meaning of value. These concepts are *Better Health*, *Better Care* and *Low Cost*. Figure 1 shows a brief schema of these concepts.

The *Better Health* concept aims to improve the general status of the patient. Better Health Systems are intended to provide the best care possible to achieve healthy populations. This concept can be related to paradigms such as Evidence-Based Medicine that seek the best evidence for selecting the best care protocols and Personalized medicine, which is more focused on finding specific protocols for specific patients.

The *Better Care* concept is related to the patient’s experience of care. This concept aims to improve the patient’s perceptions of their interaction with the health system. Also, this concept is related to the Personalized Medicine concept because, in this paradigm, by selecting the best treatments, the experience of care is taken into account. Finally, the main aim of the *Lower Cost* concept is to optimize care in order to ensure the sustainability of health systems.

The Big Data paradigm and Data Science can be used to support Value-Based healthcare delivery [3,16]. However, despite the great advantages in the application of Big Data to healthcare, health professionals are somewhat suspicious of how current expert clinical knowledge will be integrated into automatically learned clinical models [17]. So, it is necessary to use new models to incorporate this working knowledge into data science models in a better way. In this line, Interactive Pattern Recognition (IPR) [6] is a formal framework that introduces the medical expert who is engaging in the learning process and allows them to correct the hypothesis model in each iteration to prevent undesirable errors and to converge to a solution in an iterative manner. However, this framework requires human-understandable machine learning frameworks to realize these possibilities. The application of Process Mining within this framework is a promising solution that may fill this gap.

### 2.2. Process Mining in Emergency Rooms

Process Mining [7] is a research discipline that uses existing information in clinical databases and Hospital Information Systems (HIS) to create human-understandable views that support healthcare stakeholders in enhancing their understanding of the clinical process. In the last several years, Process Mining has been applied in the medical domain [18,19]. There are some applications in which the implementation of Process Mining has successfully demonstrated how medical experts can discover clinical protocols in different disciplines, such as dental treatments [20], surgery flow [21] or chemotherapy [22]. Also, Process Mining Interactive methodologies have been proposed to support the application of these technologies in the medical domain. This is the case for the Question-Driven methodology [9]. This methodology proposes the formulation of research questions that are based on the daily problems encountered by physicians and the use of Process Mining technologies to solve them.

There are some recent studies that have applied Process Mining to medical emergencies. In References [23,24], the authors applied Process Mining control-flow discovery and clustering techniques to determine the most common emergency unit flows. In Reference [25], different hospitals were compared for their adherence to triage protocols by measuring patient flows using discovery techniques. In Reference [26], the emergency flow was analyzed using a Question-Driven methodology, which is an interactive methodology [6] that is intended to support health professionals by answering their specific questions in an iterative way. In Reference [27], the first attempt to characterize processes for stroke treatment was made; the pre-hospitalization process and Stroke treatment were identified in this work but the Emergency process of stroke treatment was not characterized. In addition to discovering process flows, in order to use these techniques in stroke cases, for which timing is crucial, it is also necessary to analyze the time spent in each of the emergency stages to properly characterize and compare the processes.

### 2.3. The Emergency Process

**Figure 2:**
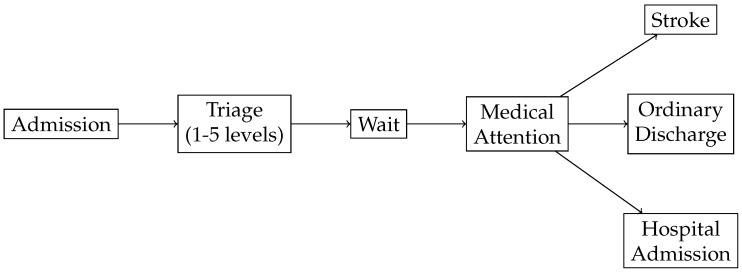
General Flow of Medical Emergencies.

In order to apply Value-based healthcare, the first step is to identify the process. Figure 2 illustrates the Emergency Process in a hospital. The process starts when a patient arrives at the hospital and is administratively *admitted*. Then, the patient waits until the clinical staff performs a *triage*. The triage step is guided by software that provides questions to be posed to the patient in order to determine the level of priority (classified into one of 5 existing levels, from lowest (5) to highest (1) priority, according to the classical Manchester codification [28]). Next, the patient *waits* until the system assigns a physician to the case on the basis of the physician’s discipline, the patient’s priority and the availability of resources (represented by the Wait node). Then, the patient receives medical *attention* and the case is discharged (represented by the Attention node). Given that this work focuses on the stroke emergency process, we distinguish three possible discharges: *Ordinary Discharge* (the patient is sent home), *Stroke* (the patient goes directly to the special unit that treats this cases) and *Hospital Admission* (to treat other complex cases that are beyond the scope of this work).

Prior to the last step, everything is ready to deliver medical attention, depending on the patient’s priority level. When a physician is free, he selects a patient in the computer system depending on his specialty and the patient’s priority. This starts the Medical Attention process that concludes with the discharge of the patient. Depending on the final assessment, the patient is identified as a Stroke case, OrdinaryDischarge or a Hospital Admission.

In Emergency Room processes, the treatment of Stroke patients is a critical case. In such cases, the accurate coordination of clinicians is crucial. Stroke is an illness that has high morbidity and mortality rates. It is especially significant because of the high sociosanitary and associated disability costs [29,30]. The potential outcomes not only have a strong impact on the quality life of patients but also can increase costs for the health system [31]. The adequate diagnosis and the timely and coordinated action of health professionals are decisive in saving the patient’s life and preventing their cognitive decline [32]. The prognosis of stroke depends largely on the ability to maximally reduce brain injury. One of the keys to success is the creation and optimization of primary care and emergency protocols for quick diagnosis and treatment to shorten the time between the stroke event and the application of adequate treatment. With this in mind, the creation of Data Science tools for the continuous analysis of primary care and emergency protocols will be a clear advantage that improves clinical processes for critical diseases such as stroke.

The aim of this paper is to evaluate how Process Mining can reveal differences in emergency processes, specifically differences in the treatment of stroke patients. Although topologically the stages followed for stroke episodes are the same as those for ordinary or other hospital admissions, it is expected that differences will be found in the time spent in some of the stages in light of the special characteristics of the problem. To show the differences in the time spent, depending on the level of emergency, we labeled the nodes of wait and attention time with the level of triage selected. Also, we labeled events with the most common discharge outcomes (Exitus (Death), Home, Primary Care, etc.).

## 3. PMApp and Statistical Significance Maps

For the application of Process Mining technologies, we used PMApp. PMApp is a Process Mining toolkit that is based on the PALIA Suite tool [21]. PMApp enables the creation of custom Process Mining dashboards specific to the Medical Domain. This framework has been successfully tested in the application of the Question-Driven methodology using emergency data [26] and it has been applied to other medical domains such as surgery [21] and diabetes cases [33]. PMApp also enables the creation of interactive dashboards that respond to the selection of arrows and nodes by capturing GUI events and it also allows the user to create custom forms and algorithms for discovery, filters, enhancement maps, and so forth. PMApp implements the PALIA algorithm [21], which results in the traceability of all learning process, so each activity is continuously associated with single events. In PMApp, it is possible to render maps that can enhance the discovered model using color gradients. With this feature, it is possible to render specific maps that highlight specific situations that depend on a customized formulation and are represented by nodes. We can use this technique to facilitate health professionals’ understanding of the processes. We can create maps for common aspects, such as performance, duration of activities, the number of cases, the number of events, and so forth but we can also create specific maps that highlight specific situations customized for the problem. This can be used to create more specific dashboards that provide medical doctors with more personalized support to increase the usability, utility and reliability of the technology.

To compile new medical evidence, the concept of statistical significance is critical. To evaluate and measure medical processes, it is necessary to show the differences among them. Although there are some suspicions toward the interpretation and use of statistical significance indexes such as the *p*-Value [34,35], it is clear from the clinical literature that most researchers have focused on measuring the statistical significance using *p*-Values [36]. In order to provide trustworthy information to healthcare professionals, it is desirable to apply a measure of statistical significance to the flow to increase the acceptance of the results generated by Process Mining algorithms in the medical domain. In order to increase the medical confidence in Process Mining results and validate the results obtained, we implemented a specific map in PMApp that highlights the activities whose duration is statistically significantly different.

**Figure 3:**
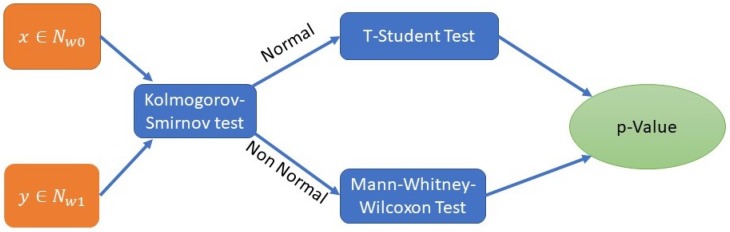
Calculation of *p*-Value in the Statistical Significance Map.

Figure 3 shows the process for evaluating the statistical significance using the *p*-Value. A pairwise comparison of nodes that refer to the same activities is performed. From PMApp, we get a set of times for each execution associated with each activity. We apply the *Kolmogorov–Smirnov Test* in order to evaluate the normality of the distribution of the time values. If the two distributions pass the normality test, we use a T-Student Test for the computation of the *p*-Value. If not, we assume that the distributions are not normal and we apply the *Mann–Whitney–Wilcoxon Test*. In both cases, if the *p*-Value is lower than a given threshold, we can assume that the distributions are significantly different. For this process, we set this threshold to 0.05, which is frequently reported in the literature.

**Figure 4:**
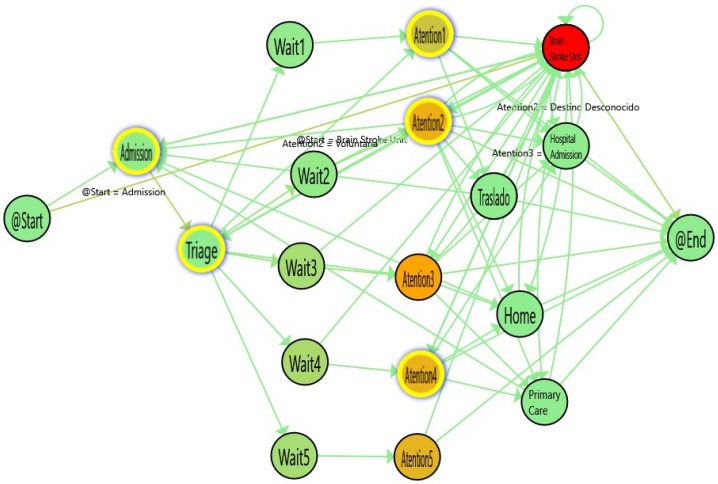
Flow of Stroke episodes with Statistical Significance Map. The colors in nodes represent the average time spent in each activity.

Figure 4 shows an application of the Statistical significance Map from a Process Mining discovery result after comparing two flows. The nodes highlighted by a yellow border are those that have *p*-Values below the threshold.

With such a map, doctors can compare the differences in each stage of the process. In this example, the gradient colors of the arrows represent the number of cases and the gradient colors of the nodes show the time spent in each stage of the Emergency process. The greener the color, the less time used to perform this activity; on the contrary, the redder the node, the more time spent in the stage. However, although these differences can be high, it is only when the statistical significance is computed that we can demonstrate these differences and acquire the trust of medical doctors. Using this process map, health professionals are able to not only evaluate the changes in the flow but also distinguish which of those changes are statistically significant. This measure will assist health professionals in the detection of differences that are supported by strong evidence. A comparison between two nodes can result in high median/average differences but a high *p*-Value. This scenario means that there is no real evidence that patient behavior at this point is actually different. On the contrary, if the *p*-Value is lower than the threshold (typically 0.05), then it is assumed that there are compelling reasons to believe that the behavior of the patients at that point of the flow is different. This information is crucial to the discovery and demonstration of medical evidence.

## 4. Experiments

In this work, we used real data from the emergency flow of 2145 Stroke patients who were treated between January 2010 and June 2017. The log information was acquired from the Hospital Information System (HIS) with a timestamp granularity of seconds.

**Figure 5:**
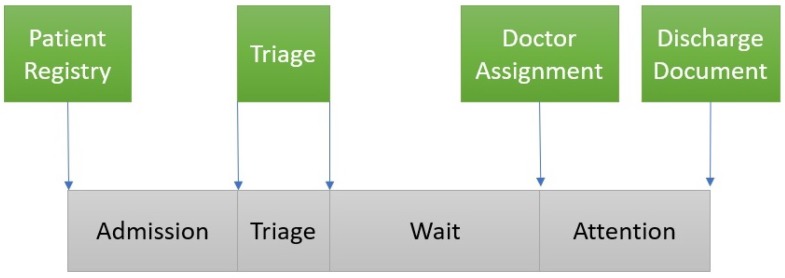
Log extraction from hospital computerized applications.

The Figure 5 shows how the log is captured from Hospital Databases. Each activity in the log is extracted from the timestamps of the computerized administrative tools that are currently installed in the emergency unit. From the patient Registry application we extract the timestamp of the registration of the patient. This starts the Admission activity until the triage. From the Triage application tool, we extract the timestamp of the start and the end of the triage process. When the doctor is ready, selects a patient of the waiting list in the application that produces the timestamp that start the attention, finishing in the moment when the physician signs the discharge document.

**Figure 6:**
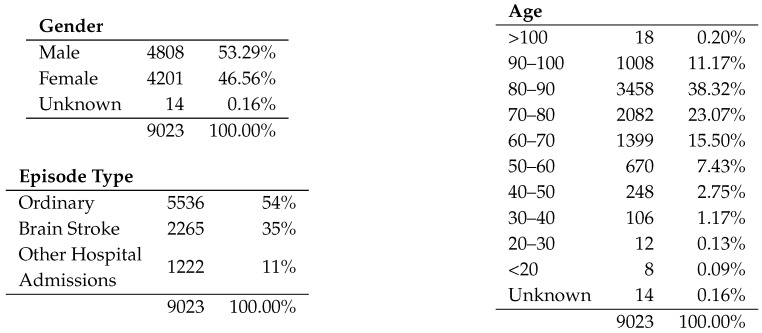
Log stats.

Figure 6 shows the general stats of the log. In this log, we set 9046 emergency episodes that can be divided into three kinds of cases depending on the discharge outcome: 5536 (54%) are Ordinary episodes, 2265 (35%) correspond to Stroke episodes and 1222 (11%) are episodes with a hospital admission for reasons that are not directly related to stroke. In these stats, it is possible to see an adequate gender balance as well as an age distribution that, as noted in classical studies, shows that the prevalence of brain stroke patients is higher in elderly people.

In order to prepare the data to be used for the Process Mining Log, we made some assumptions.

- The database does not directly show a connection between admission to the Brain Stroke Unit and the emergency episode. Therefore, we assumed that if an Emergency episode occurred 24 h before admission to the Brain Stroke Unit, the two events are related. For that these activities will be grouped in the same trace.
- The admission entry information available in the HIS includes the date but not the time of admission. Therefore, we assumed that the admission hour of the hospital corresponds with the discharge from the Emergency Unit.
- The database does not show a connection between different emergency visits for the same episodes. Therefore, we assumed that emergency visits separated by less than 24 h represent the same episode.For that these activities will be grouped in the same trace.
- Extremely urgent patients undergo bureaucratic admission after they receive medical attention and triage is usually performed prior to the arrival at the hospital in an ambulance. This makes the time of admission non-representative of the real process. In these cases, we assumed that the admission and triage were performed just prior to the attention stage (when a medical doctor is assigned to the patient in the HIS) and that their duration is 0.

Using this log, we evaluated the proposed questions using Process Mining Techniques.

### 4.1. Q1: Can Process Mining Detect and Measure the Special Characteristics of Stroke Emergency Processes?

In order to deploy a successful protocol in healthcare, it is not only necessary to have adequate clinical knowledge but also crucial to detect the specific characteristics of the protocols implemented. Usually, problems such as variability in the behavior of health professionals and the logistic problems in health systems have an impact on the deployment of policy in a real health system. In order to prevent, as much as possible, the discrepancies between the designed and the real process, it is important to discover the special characteristics of the process. This knowledge can support health experts in the design of adequate policies.

Our hypothesis underlying this question is that Process Mining is a technology that enables experts to understand the actual processes being implemented. In our context, the question is intended to show how health experts can take advantage of Process Mining technologies to, on the one hand, identify the process of emergency treatment and, on the other hand, discover the differences in a special process such as the stroke emergency. Although the emergency process for stroke episodes should be similar to that for other emergency cases, the time spent in each stage should vary.

**Figure 7:**
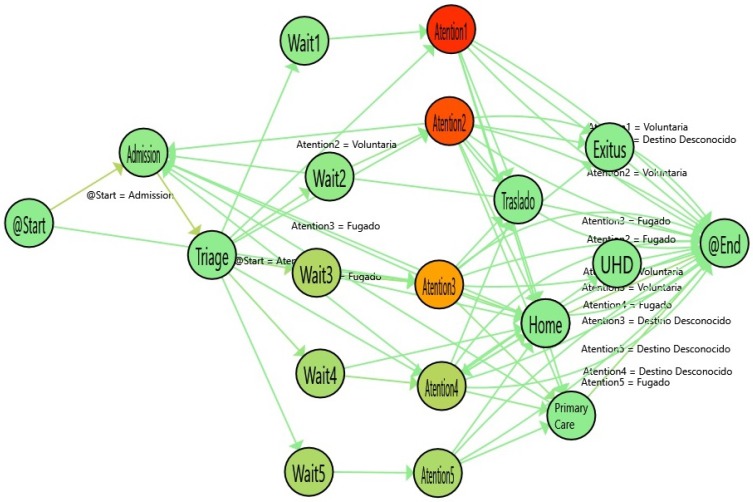
Flow of the ordinary discharge episodes for Q1. The colors in the nodes represent the average time spent in each activity.

In Figure 7, the process for ordinary cases are shown. The color gradient in each stage represents the time spent. In this line, the redder the node, the more time spent on the activity. This enhancement of the flow can be used to illustrate the special characteristics of the ordinary emergency process. In that case, the waiting time is higher, depending on the emergency priority. Thus, the higher the priority of a patient in triage, the less the waiting time. On the contrary, patients who have more serious illnesses spend more time in the attention stage compared with cases that are not urgent. As expected, the most complex illnesses need more time to be treated.

The simplicity of the models allows a better understanding of the clinician, that provides a quick view of the process. It is important to notice that, not only the nodes but also the paths also provide interesting information. For instance, the incoming flows to Admission are patients that have returned to emergencies in less than 24 h. In Emergency domain, it is considered that if a patient returns in less than 24 h is the same episode. In fact, a common performance indicator in emergencies is the re-admissions in less than 24 h (Sometimes 72, or even in some cases, one month). In that case, the incoming flows to admission are the re-admissions suffered by the patient. So, the differences in the paths are due to these differences in the re-admissions.

In Figure 4, the flow for stroke patients is shown. Table 1 shows the numerical information derived from the statistical comparison between Stroke and Ordinary Patients. The nodes with the highlighted borders represent a statistically significant difference in the duration of these activities. In general, the waiting times are similar. However, the Admission and the Triage times are statistically significantly lower for stroke patients and the Attention Time is lower for urgent levels (1 and 2) and higher for non-urgent levels (4 and 5). As expected, the differences in the high-priority emergencies show that the patients who are quickly diagnosed with Stroke are immediately sent to the stroke unit, so they spend less time receiving attention in the emergency area. On the other hand, data on low-priority emergencies show that when the Stroke is not quickly diagnosed, the time spent receiving attention is significantly increased (up to 8.67 times worse than ordinary episodes in level 4).

### 4.2. Q2: Is Process Mining Able to Measure Organizational Changes in the Stroke Emergency Process?

Once the process is identified, in order to provide optimal care, it is necessary to evaluate the application of policies to assess the value delivered to the patient. Usually, it is not easy to evaluate the effects of a policy on the process. However, using Process Mining technologies, it is possible to evaluate the impact of a policy on an organization by comparing the identified base process with the process discovered after the application of the policy. Without an adequate and timely evaluation process, an organizational change that is perceived positively by healthcare policymakers could actually have negative effects on the organization. In our previous question (Q1), the answer is based on discovering the basic characteristics of the stroke process. Our hypothesis is that this discovered process can be used as a basis for analyzing behavioral differences. In that case, comparing the behavior of the process before and after the organizational change can provide an interactive view to policymakers that enables them to identify and quantify the impact of policies on the process.

After a previous study, the managers of an Emergency Room area decided to establish a second triage station in March 2017. The objective was to improve the time of admission to ensure a good quality of service in the most complex emergencies. The objective of this question is to evaluate how Process Mining can identify the effects of this change on the treatment of complex illnesses, such as Stroke. In order to perform the study, we selected all the stroke episodes between January and June of 2017. With those events, we created two logs: The single-triage log (before March) and the double-triage log (after March). The single-triage log is formed by 284 (40%) episodes and the double-triage log is formed by 425 (60%) episodes.

**Figure 8:**
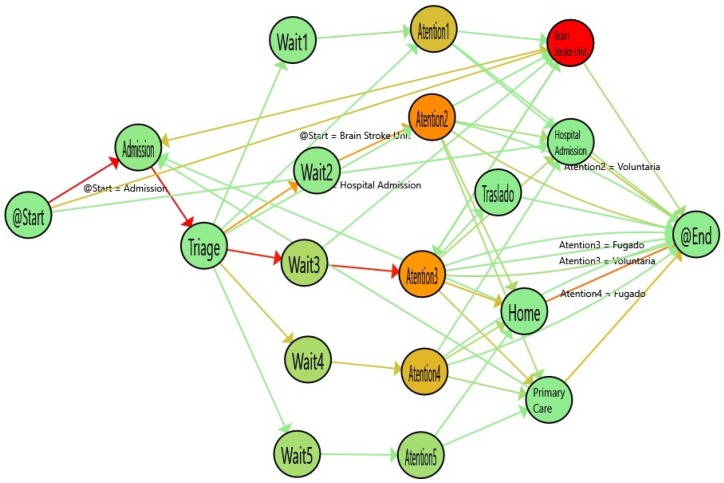
Flow for single triage (from January to March 2017). The colors in the nodes represent the average time spent on each activity and the colors of the edges represent the number of patients that followed this path.

**Figure 9:**
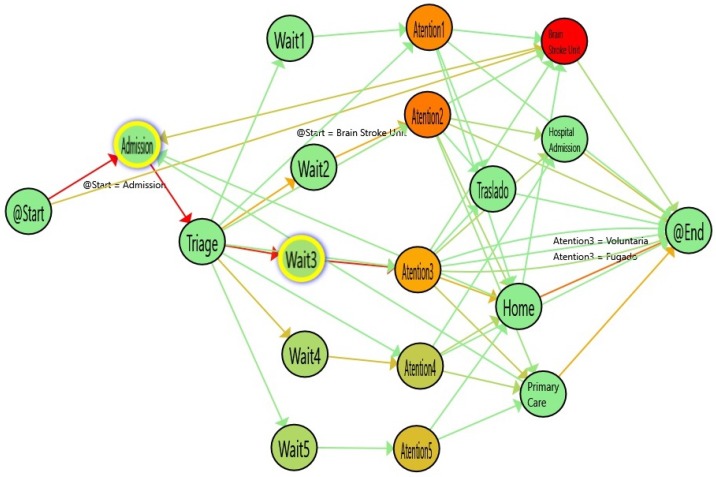
Flow for double triage (from March to June 2017). The colors in nodes represent the average time spent on each activity and the colors of the edges represent the number of patients that followed this path.

Figure 8 shows the results of analyzing the single-triage log and Figure 9 shows the flow inferred from the double-triage log. Table 2 shows the stats associated with the analyzed logs. In Figure 9, the nodes with the highlighted borders represent the activities with a duration that is statistically significantly different. By looking at these nodes, it is observed that the admission activity has a significant decrease of 3.26 min (30% improvement). Also, the time for the Wait3 Node is significantly decreased by 16 min (28% improvement). The Wait3 node is the most commonly selected triage. This reduction in time suggests that Wait3 patients have a significantly reduced risk by being treated in less than one hour. Administering treatment to stroke patients in less than one hour is critical because it reduces the risk of cognitive impairment, increases the probability of rehabilitation and reduces the risk of developing associated comorbidities and, ultimately, the sanitary costs that result from them.

### 4.3. Q3: Can Process Mining Reveal Differences in Emergency Process Protocols Depending on Patients’ Personal Characteristics?

In healthcare, because human behavior is variable, the impact of policies does not produce the same effects on all patients. The actuation of a policy that has a positive impact on a specific group of patients can have no effect on others or even have a negative impact on the process. In such cases, it is necessary to provide the appropriate tools to ensure a detailed evaluation of the policies or identify specific risk groups in the healthcare process. The goal of investigating the current question is to identify differences in the process that do not depend on the process itself but on the specific behaviors of patients.

In Emergency Rooms, the age of a patient affects the management of the illness. To answer this question, we analyzed the behavioral differences on the basis of the age of the patients in the emergency process. In order to do that, we categorized the data from the whole log (Ordinary, Entry, and Stroke) stratified according to the age of the patients. Table 3 shows the distribution of the age groups.

**Figure 10:**
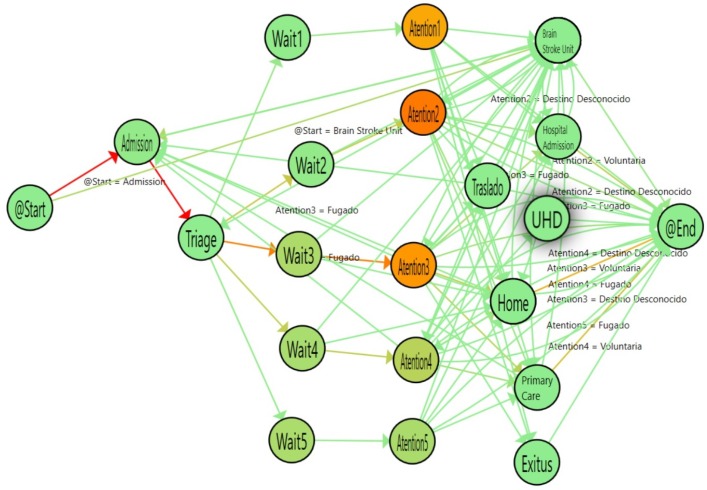
Emergency Room flow determined for patients aged 65+ years.

**Figure 11:**
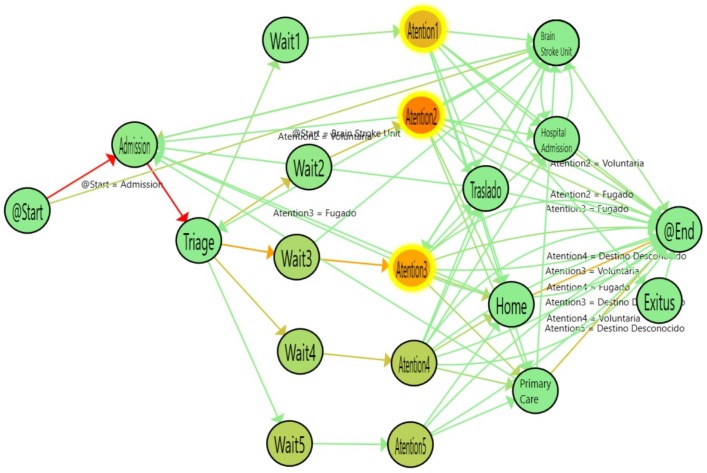
Emergency Room Flow determined for patients from 40 to 65 years old. Highlighted Nodes indicate a statistically significant difference compared with patients age 65+ years.

Figure 10 shows the flow followed by older adults (aged 65 years or more). Figure 11 shows the flow of adult patients (between 40 and 65 years old) and Table 4 shows the statistical results of comparing the logs. According to the results obtained, the waiting time for adults is similar to that for older adults. However, there is a significant difference in the time spent at the attention stage for the most urgent levels (1, 2 and 3) between the adult and older adult groups, with differences of less than an hour in the three cases.

**Figure 12:**
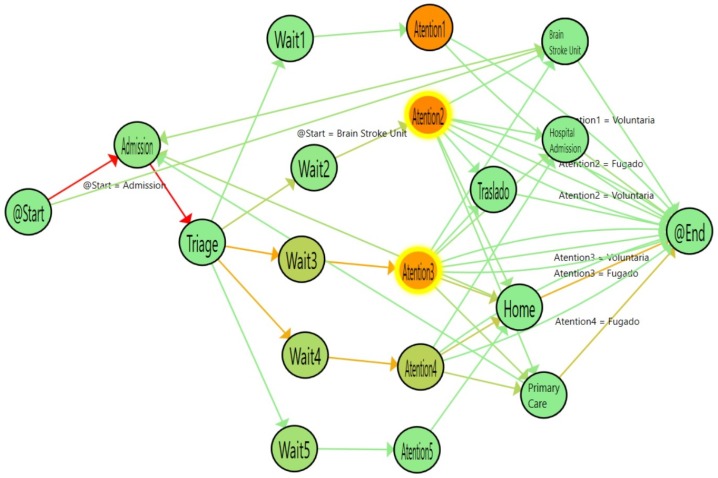
Emergency Room Flow discovered for patients from 20 to 40 years old. Highlighted Nodes show statistical significance in comparison with patients aged 65+ years.

In a similar way, we compared Older adults with young patients (20–40). Figure 12 shows the flow inferred from the data and Table 5 shows the numerical results of the comparison between the two groups. The waiting time is similar between the groups and the Attention time is significantly different for the level 2 and 3 urgency patients, who have a much different median time, with differences of up to two hours for young patients compared with older patients.

### 4.4. Q4: Can Process Mining Evaluate the Status of the Emergency Protocol According to Existing Gold Standards?

The aim of this question is to determine whether Process Mining can support Emergency Room health professionals in the evaluation of their protocols. Behind the concept of Evidence-Based Medicine is an aim to provide recommendations and guidelines to health professionals to improve the daily practice protocols according to the evidence acquired from medical research. One of these recommendations is the Manchester Standard of Triage [37]. This protocol is a set of recommended timelines for triage waiting times, depending on the seriousness of the illness. According to this standard, urgent patients should be attended to quicker than non-urgent patients but the waiting times of non-urgent patients should be bounded.

Table 6 shows the recommended maximum waiting times (in minutes) for each urgency level according to the standard. The recommendations are that level 1 patients should be attended to immediately and level 5 patients can wait for up to 240 min. Manchester standard does not offer recommendations for the attention time To simplify the view of the fulfillment of these recommendations, we propose the creation of a color-gradient map that is based on these times. The idea is to use colors to show the adequacy (or lack thereof) of the timing of the process according to the Manchester triage standard. In Figure 6, the gradient range reflects the range selected, which goes from 0 to twice the time recommended by the Manchester Triage Standard. The Green to Yellow color gradient is used to illustrate enhancement and reflects that the waiting times meet the standard, with yellow representing the upper limit of the Manchester recommendations. On the other hand, the redder the color, the more the waiting time exceeds the hour limit.

The factor to compute the gradient was normalized using the median of the times spent in each activity, depending on the level. Formally, the factor $M_{level}$ is computed according to the following equation:

(1) $$M_{level}=\frac{Median(Durations_{level})}{Factor_{level}*2}$$

**Figure 13:**
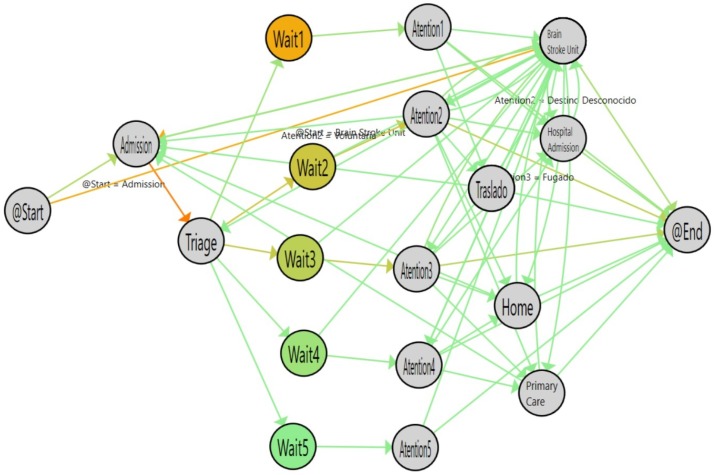
Adequacy of treatment of Stroke patients according to the Manchester Standard.

Figure 13 shows the Manchester enhancement map of the flow discovered from analyzing cases of stroke patients. As can be seen, the only red color is for level 1. Levels 2 and 3 are darker but still within the time limit. It is important to note that for level 1 patients, who require immediate attention, the waiting times can be erroneously recorded because of the urgency and priority to take care of the patient before addressing administrative tasks. Because of these cases, the imprecision in the data can depict deviations in the color gradients that do not reflect the real situation. Numerical data are shown in Table 7.

**Figure 14:**
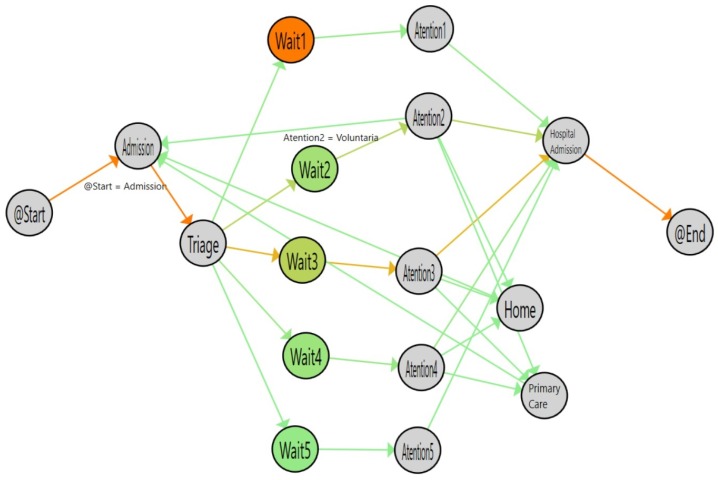
Adequacy of the Admission of patients according to the Manchester Standard.

Figure 14 shows the Manchester enhancement map of the Admission Log. As can be seen, Level 1 has deviations from the Stroke Log and, on the contrary, waiting times for level 2 are less.

**Figure 15:**
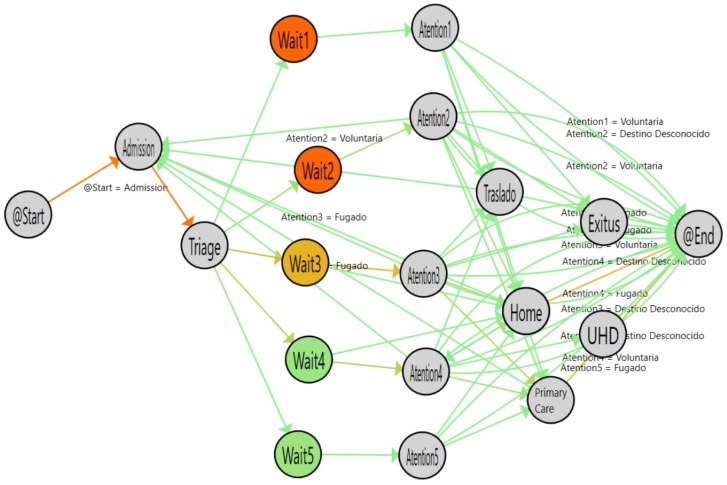
Adequacy of treating Ordinary patients according to the Manchester Standard.

Figure 15 shows the Manchester enhancement map of the Log for ordinary patients. The results show more deviation in these cases than in the previous cases.

### 4.5. Q5: Can Process Mining Provide a Healthcare Value-Based View of the Effects of the Care Provided?

The main objective of Value-based Healthcare is to maximize the value transferred to the patient with the resources used. The aim of emergency rooms is not to completely heal the patient; Emergency Rooms are intended to resolve the Emergency event by stabilizing the patient within a time that is as short as possible. Sometimes, Emergency events are not entirely resolved and the patient returns to the hospital Emergency Room. This is the concept of *Readmission* [38]. Usually, when the patient returns to the Emergency Room for treatment in less than 72 h, the patient’s original episode is considered unresolved. So, in Emergency Rooms, the number of readmissions is a measure of the quality of care provided to the patient and, in turn, a Value-based measure for the evaluation of the care provided to the patient.

**Figure 16:**
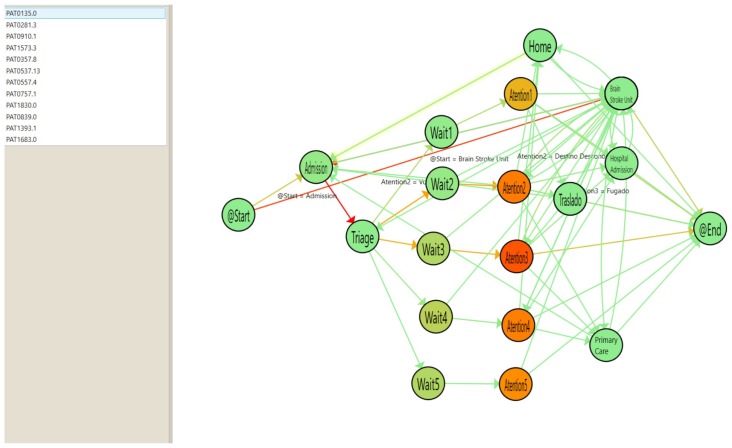
List of patients Readmitted after a Home Discharge.

By creating a PMApp filter that fuses episodes occurring within less than 72 h of each other, we can illustrate the flow to easily show the episodes that return for admission after a discharge. Using color gradients in the arrows, Health professionals can view the number of readmissions by discharge type. In Addition, PMApp enables complete traceability of associated events with arrows and nodes. Figure 16 shows readmissions from the Home state to the Admission state by the arrows between these two nodes.

**Figure 17:**
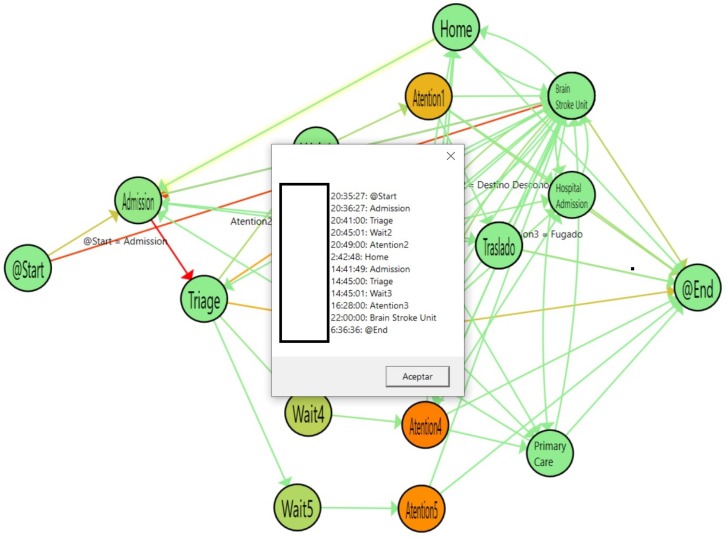
Individual flow for Readmitted patients.

Also, by clicking any of the cases on the list, we can create a summary of the individual patient with the listed set of events that this individual patient has experienced. An example is shown in Figure 17.

## 5. Discussion

The application of value-based healthcare concepts in real scenarios requires information that should be processed and presented in an understandable way to health professionals. Classical Data Mining technologies are becoming black boxes for health professionals who have not had the opportunity to understand the Why of the results obtained from these Intelligent systems. The use of human-understandable automatic learning techniques will effectively solve the black box problem.

In this paper, we present our analysis of the application of Process Mining tools for supporting Health professionals in their implementation of value-based delivery of care. These questions were designed to show how the Value-based Healthcare and Triple Aim Paradigms can be approached in specific Emergency Room cases.

**Figure 18:**
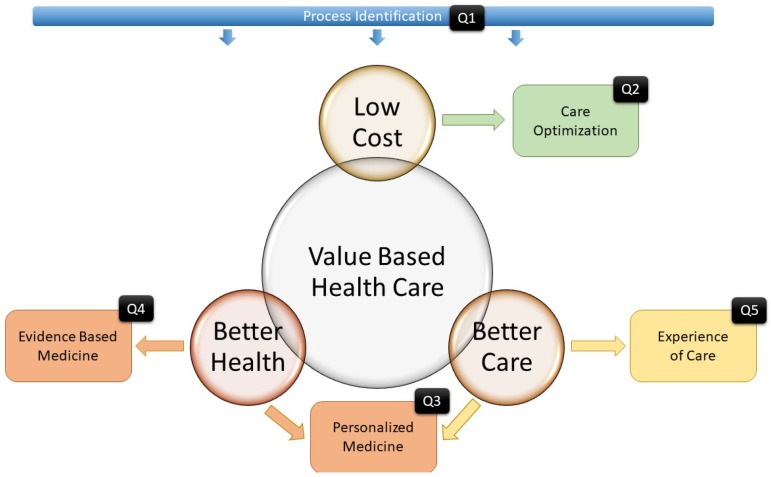
Value-based Healthcare and Triple Aim paradigms related to the research questions.

We do not claim that the presented study is exhaustive. A complete and systematic set of techniques that covers all the stages of Emergency Room Services will need to greatly expand on the current study and this is beyond the scope of this work. Our study points out some ideas to support Value-Based Healthcare in Emergency Rooms for patients, including critical cases such as Stroke, using the existing data in the Hospital Information System. Figure 18 summarizes the research design of the questions. Question 1 is intended to demonstrate that Process Identification is the basis for analyzing the rest of the questions. Question 2 shows how Interactive process mining can be used to improve patient care by analyzing whether the value realized by organizational changes is worth the required dedicated resources. Question 3 evaluates how Process Mining techniques can be used to reveal the differences between groups of patients or even between individual patients and it emphasizes personalized care and better patient health. Question 4 aims to compare, via delta analysis, the execution of clinical protocols with existing knowledge-driven clinical evidence. This enables not only a quantitative but also a qualitative evaluation. Finally, Question 5 proposes a way to not only evaluate the quality of the care delivered to patients but also detect anomalous readmissions and provide support for case-by-case assessments by health professionals.

This study also takes into account the idiosyncrasy of the medical research field and incorporates the concept of statistical significance by creating specific enhancement maps using the widely used *p*-Value. The measure of statistical significance is crucial for achieving the trust of medical practitioners. In the clinical world, if the results are not supported by an evaluation of the statistical evidence, we cannot trust them as actual medical evidence. Although, there are different ways of compare differences between cohorts of patients, in this paper the statistical significance is mainly focused in detecting differences in the time spent in the activities. However, it is possible to discover many variations in the flows by showing the differences in the arcs between the activities.

From a more clinical aspect, we formally report the differences between the Ordinary, Admission and Stroke processes in the Emergency Room scenario. We observed that the stroke emergency process has clear differences in the time spent in the triage, admission and attention stages. The stroke process requires a specific treatment that should be covered by the stroke unit of the hospital and the emergency physicians should stabilize and send the patient to the unit as soon as possible. In this process, triage is crucial and the selection of the correct emergency level significantly decreases the time of stay in emergencies. In our study, we detected a set of under-triaged stroke patients who were incorrectly classified and this is probably caused by the confusion associated with a typical level 4 disease. Misclassification dramatically increases (by 867%) the time in the attention stage. Also, we discovered how the use of an additional nurse working in triage can save crucial minutes that can be decisive for the survival or cognitive decline of the patient. We show how the age of patients affects the time spent in the attention stage in the Emergency Room. Specifically, elderly people may spend over 2 h longer than young people in the attention stage. Further, we state that critical illnesses, such as stroke, are more adequate according to the triage standard compared with ordinary illnesses.

One of the main contributions of this work is the integration of statistical significance tests into process mining tools. By incorporating this feature we are confident that clinical and biomedical professionals will be able to see its benefits and power on daily basis medical practice. However, this novel contribution has some drawbacks which should be considered in future works. Both the size of the sample data and the size of the effect we want to test are factors that affect if the differences observed in the times are not attributable to chance (that is, are statistically significant). For instance, a difference of 5 s is not comparable as as a difference of 5 min in the waiting time. In the experiments reported hereby we yielded a sample size enough to have a high statistical power and moreover, the effects on time are of consideration. Future developments should also foresee the limitations imposed by multiple testing (doing tests on many variables in the same experiment), since the probability of success is reduced in proportion to the number of tests and the Bonferroni correction should be applied

These are some findings that clinicians can achieve using Process Mining technologies in their research. This technology can support health professionals aiming to implement the value-based delivery of care by making the process easier. This enables health actors to not only optimize the care process but also to practically apply the paradigms that lead to better care and better health.

## 6. Conclusions

In this paper we have analized how Interactive Process Mining technologies can support the application of new medical paradigms like Value-based Healthcare and Triple Aim. This study has shown how Process mining can identify the processes in health services like emergencies: characterizing the specifity of critical illnesses like Stroke; evaluating and measuring how affects organizational changes to the process, discovering differences in the behavior of patients; comparing the actual process with the gold standards, and show the chain-value of the process to the patient.

These indicators are being applied in a real hospital and are used by health professionals in an interactive way, achieving very promising results.

## Figures and Tables

**Table 1:** Sample size and descriptive statistics for the time (in minutes) for Ordinary and Stroke Unit Admission Nodes. Bold rows are for statistically significant differences between groups.

Ordinary Emergency			Stroke Emergency		
**Activity**	N	IQRange	N	IQRange	***p*** **-Value**
**Admission**	**5630**	**9.27 [4.62, 18.53]**	**1475**	**7.12 [4.13, 13.80]**	**0.00**
**Triage**	**5630**	**1.00 [0.00, 2.00]**	**1475**	**1.00 [0.00, 2.00]**	**0.05**
Wait1	41	7.97 [2.97, 15.47]	126	4.97 [2.72, 8.97]	0.13
**Attention1**	**53**	**389.50 [208.82, 667.47]**	**180**	**110.82 [74.60, 213.07]**	**0.00**
Wait3	2960	53.47 [21.97, 110.97]	555	36.97 [12.97, 83.97]	0.07
Attention3	3016	220.56 [128.48, 355.56]	576	247.07 [152.45, 373.81]	0.72
Wait2	829	7.97 [4.97, 15.97]	613	7.97 [3.97, 16.97]	0.83
Wait4	1571	51.97 [22.97, 103.97]	43	61.97 [23.97, 121.97]	0.62
**Attention4**	**1590**	**27.68 [10.54, 99.55]**	**43**	**240.15 [116.78, 384.62]**	**0.00**
**Attention2**	**866**	**305.53 [195.90, 568.57]**	**673**	**210.62 [133.84, 304.88]**	**0.00**
Wait5	105	73.97 [34.97, 116.47]	3	3.97 [0.97, 185.97]	0.77
Attention5	105	25.17 [9.22, 68.44]	3	86.30 [54.05, 563.27]	0.45

**Table 2:** Sample size and descriptive statistics for the time (in minutes) for Ordinary and Stroke Unit Admission Nodes. Bold rows are for statistically significant differences between groups.

Single Triage			Double Triage		
**Activity**	N	IQRange	N	IQRange	***p*** **-Value**
**Admission**	**284**	**11.01 [4.71, 24.27]**	**425**	**7.75 [3.53, 17.96]**	**0.00**
Triage	284	2.00 [2.00, 4.00]	425	2.00 [1.00, 4.00]	0.29
Wait5	3	26.97 [24.97, 151.97]	7	73.97 [20.97, 157.97]	0.66
Attention5	3	25.13 [9.62, 141.52]	7	56.68 [33.78, 254.33]	0.27
Wait2	85	6.97 [3.97, 12.47]	108	6.97 [4.97, 13.97]	0.44
Attention2	88	242.48 [170.38, 399.66]	119	275.58 [195.95, 497.25]	0.48
**Wait3**	**142**	**56.47 [21.97, 128.22]**	**210**	**40.47 [15.97, 79.47]**	**0.00**
Attention3	142	222.09 [123.20, 410.48]	216	209.33 [124.15, 344.74]	0.35
Wait4	43	51.97 [22.97, 110.97]	74	59.97 [25.72, 107.22]	0.92
Attention4	43	63.42 [22.18, 255.33]	76	36.48 [13.51, 190.50]	0.19
Stroke	63	8640 [5760, 14400]	90	8640 [5760, 13305]	0.56
Wait1	7	7.97 [2.97, 10.97]	4	6.97 [4.22, 10.47]	0.69
Attention1	8	112.41 [47.95, 314.73]	7	149.57 [63.35, 563.50]	0.33

**Table 3:** Age groups in Q3.

Age Group	N	%
65+	6624	80.88%
40–65	1446	17.66%
20–40	113	1.38%
0–20	7	0.09%

**Table 4:** Analysis of Statistical Significance between the 65+ and 40–65 age groups (Interquartile range in Hours). Bold rows indicate statistical significance.

65+			40–65		
Admission	6744	0.14 [0.07, 0.29]	1482	0.14 [0.07, 0.29]	0.48
Triage	6744	0.02 [0.00, 0.03]	1482	0.02 [0.00, 0.03]	0.39
Wait2	1513	0.13 [0.07, 0.27]	353	0.12 [0.07, 0.23]	0.55
**Attention2**	**1513**	**4.72 [3.05, 8.16]**	**353**	**4.12 [2.72, 6.69]**	**0.00**
Wait1	202	0.05 [0.00, 0.13]	72	0.05 [0.00, 0.13]	0.96
**Attention1**	**202**	**3.02 [1.46, 6.81]**	**72**	**2.46 [1.36, 4.09]**	**0.03**
Wait3	3661	0.77 [0.30, 1.73]	662	0.80 [0.33, 1.67]	0.70
**Attention3**	**3660**	**4.06 [2.52, 6.68]**	**662**	**3.31 [1.68, 5.64]**	**0.00**
Wait4	1283	0.83 [0.38, 1.70]	372	0.88 [0.33, 1.77]	0.66
Attention4	1283	0.54 [0.19, 2.06]	372	0.42 [0.15, 1.74]	0.27
Wait5	85	1.08 [0.49, 1.84]	23	1.38 [0.75, 2.53]	0.11
Attention5	85	0.52 [0.16, 1.22]	23	0.28 [0.16, 1.41]	0.49

**Table 5:** Analysis of Statistical Significance between the 65+ and 20–40 age groups (Interquartile range in hours). Bold rows indicate statistical significance.

65+			20–40		
Admission	6744	0.14 [0.07, 0.29]	127	0.14 [0.08, 0.23]	0.13
Triage	6744	0.02 [0.00, 0.03]	127	0.02 [0.00, 0.02]	0.11
Wait2	1513	0.13 [0.07, 0.27]	21	0.13 [0.09, 0.22]	0.58
**Attention2**	**1513**	**4.72 [3.05, 8.16]**	**21**	**2.34 [1.49, 4.63]**	**0.04**
Wait4	1283	0.83 [0.38, 1.70]	47	0.57 [0.23, 1.28]	0.12
Attention4	1283	0.54 [0.19, 2.06]	47	0.36 [0.19, 0.66]	0.16
Wait3	3661	0.77 [0.30, 1.73]	53	0.98 [0.46, 1.58]	0.93
**Attention3**	**3660**	**4.06 [2.52, 6.68]**	**53**	**2.72 [0.43, 4.11]**	**0.01**
Wait1	202	0.05 [0.00, 0.13]	4	0.07 [0.00, 0.20]	0.95
Attention1	202	3.02 [1.46, 6.81]	4	3.85 [1.34, 6.35]	0.66
Wait5	85	1.08 [0.49, 1.84]	2	0.65 [0.22, 1.08]	0.36
Attention5	85	0.52 [0.16, 1.22]	2	0.13 [0.12, 0.14]	0.50

**Table 6:** Urgency levels and expected waiting times according to the Manchester Standard of Triage [37] (in minutes) and the range defined for the Gradient Map for Process Mining enhancement.

Level	Manchester Time Factor	Gradient Range
1	0	[0–2]
2	10	[0–20]
3	60	[0–120]
4	120	[0–240]
5	240	[0–480]

**Table 7:** Waiting times in the Logs (in minutes).

Level	Stroke	Admission	Ordinary
1	1.47	2.97	7.97
2	8.97	2.97	23.97
3	39.47	35.97	68.47
4	29.97	24.97	15.97
5	0.97	21.97	44.97
